# Supplementary material for: High-density volumetric super-resolution microscopy
Source: Nat Commun. 2024 Mar 2;15:1940. doi: 10.1038/s41467-024-45828-5 (PMC10908787; doi:10.1038/s41467-024-45828-5)
Supplement: Supplementary file 1 — Supplementary Information [file 41467_2024_45828_MOESM1_ESM.pdf]

Supplementary Information: High-density volumetric super-resolution microscopy

Sam Daly[a], João Ferreira Fernandes[b], Ezra Bruggeman[a], Anoushka Handa[a], Ruby Peters[c], Sarah Benaissa[d], Boya Zhang[d], Joseph S. Beckwith[a], Edward W. Sanders[a], Ruth R. Sims[e], David Klenerman[a], Simon J. Davis[b], Kevin O'Holleran[d], and Steven F. Lee[a,✉]

[a]Yusuf Hamied Department of Chemistry, Lensfield Road, University of Cambridge, Cambridge, CB2 1EW, UK
[b]Radcliffe Department of Medicine and MRC Human Immunology Unit, John Radcliffe Hospital, University of Oxford, Oxford, OX3 9DU, UK
[c]Department of Physiology, Development and Neuroscience, University of Cambridge, Cambridge, CB2 3EL, UK
[d]Cambridge Advanced Imaging Centre, Downing Site, University of Cambridge, Cambridge, CB2 3DY, UK
[e]Wavefront-Engineering Microscopy Group, Photonics Department, Institut de la Vision, Sorbonne Université, INSERM, CNRS, Institut de la Vision, Paris, France

Contents

1 Single Molecule Light Field Microscopy 3
1.1 Optical setup . . . 3
1.2 SMLFM design parameters . . . 5
1.3 Improving contrast in SMLFM . . . 5

2 Realistic 3D-SMLM Simulations 7
2.1 Engineered PSF simulations . . . 7
2.2 Light field simulations . . . 7
2.3 Detection noise model . . . 8
2.4 Simulation parameters . . . 8

3 Cramér–Rao Lower Bound 9

4 Analysis of Simulated Data 10
4.1 Defining density . . . 10
4.2 Optimising pixel size . . . 10
4.3 Defining speed improvement . . . 10

Supplementary Note 1: Single Molecule Light Field Microscopy

1.1 Optical setup

The visual representation of the platform for single-molecule light field microscopy (SMLFM) in Fig. 1 is intentionally conceptual and reduced to key components. Here we present the optical platform in more detail.

The optical set-up is based on a Fourier light field microscope, in which a microlens array (MLA), placed in the conjugate back focal plane (BFP) of a widefield microscope, locally apertures the wavefront and generates an image at the detector (Supplementary Fig. 1a) [1, 2]. This results in a single 2D snapshot where the 3D position of single-molecule fluorescence is encoded via parallax (Supplementary Fig. 1b). The hexagonal MLA (photographed in Supplementary Fig. 1c) was specifically designed to fit within the conjugate BFP to prevent partial illumination of the MLA (see Supplementary Fig. 1d and for further description Section 1.2). Partial lenslet illumination leads to distortions, such that the PSF could no longer be approximated by a 2D Gaussian function [1]. The hexagonal MLA also acts as a tube lens to focus light onto different regions of the detector. Therefore all perspective views can be approximated by a 2D Gaussian function. As a result, the position of a point emitter in each perspective view is displaced in proportion to the average gradient of the wavefront (Supplementary Fig. 1e), resulting in a point spread function comprising a hexagonal array of spots over an 8 µm axial range (Supplementary Fig. 1f).

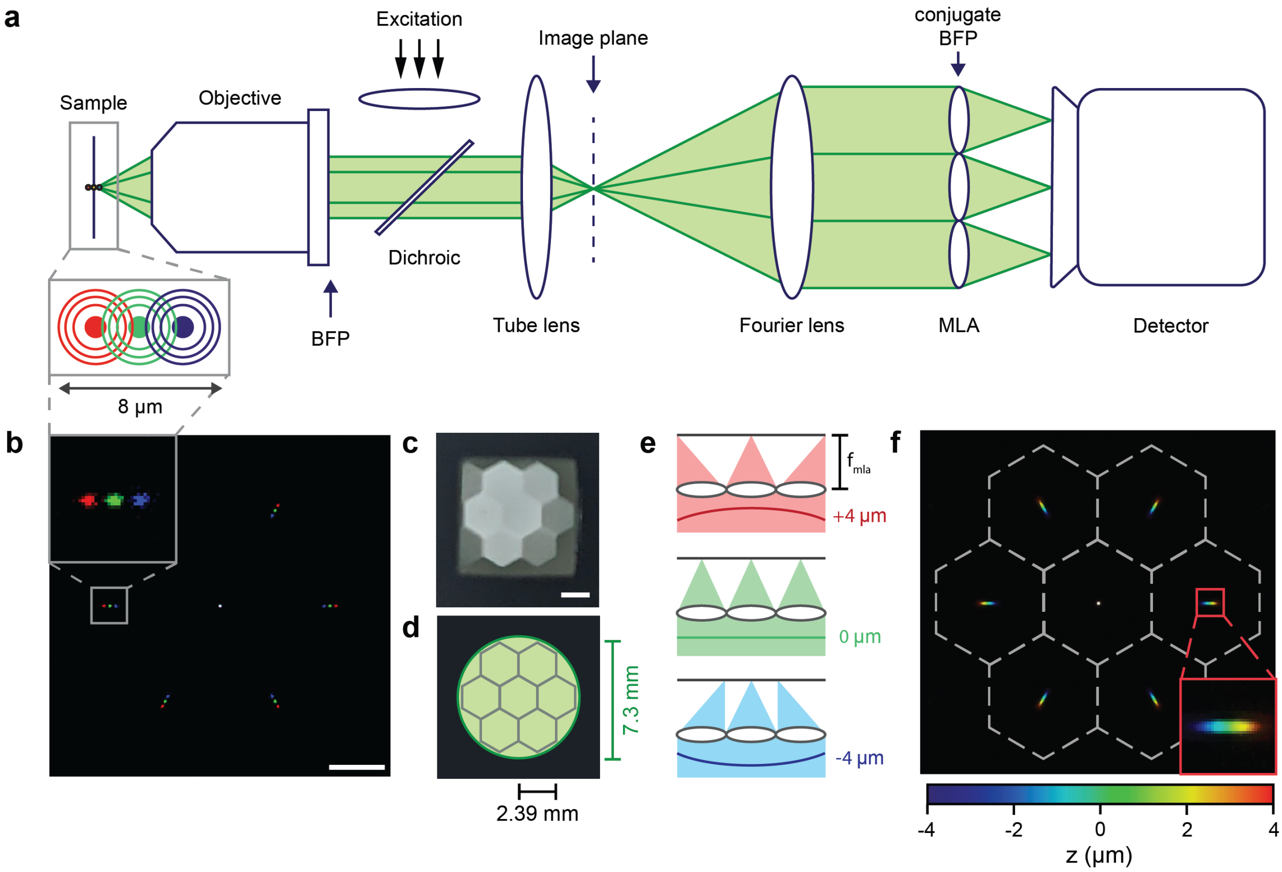

Supplementary Figure 1. The single molecule light field microscopy (SMLFM) platform used in this study. a The optical layout showing a microlens array (MLA), placed in a '3f' arrangement, segmenting the back focal plane (BFP) resulting in an image of the sample volume **b** observed from 7 different perspective views. Scale bar is 20 µm. **c** Photograph of the hexagonal MLA (scale bar 2 mm) and **d** the relative size of the MLA and the BFP. **e** The MLA samples the curvature of the wavefront and displaces the focused spot in the outer perspective views (disparity) in proportion to the depth of the emitter. **f** The point spread function (simulated) of the SMLFM platform presented herein over an 8 µm depth of field.

In this study, the performance of SMLFM is compared to other 3D-SMLM techniques. PSF engineering techniques, like the double helix PSF and tetrapod PSF, are achieved by placing a phase mask in the Fourier plane of a 4f optical relay to induce a change in the PSF as a function of depth. In the case of SMLFM, a MLA is used and phase modification is therefore combined with the image-forming lens (or tube lens) in a '3f' optical configuration. Typical optical layouts for each technique are presented in Supplementary Fig 2.

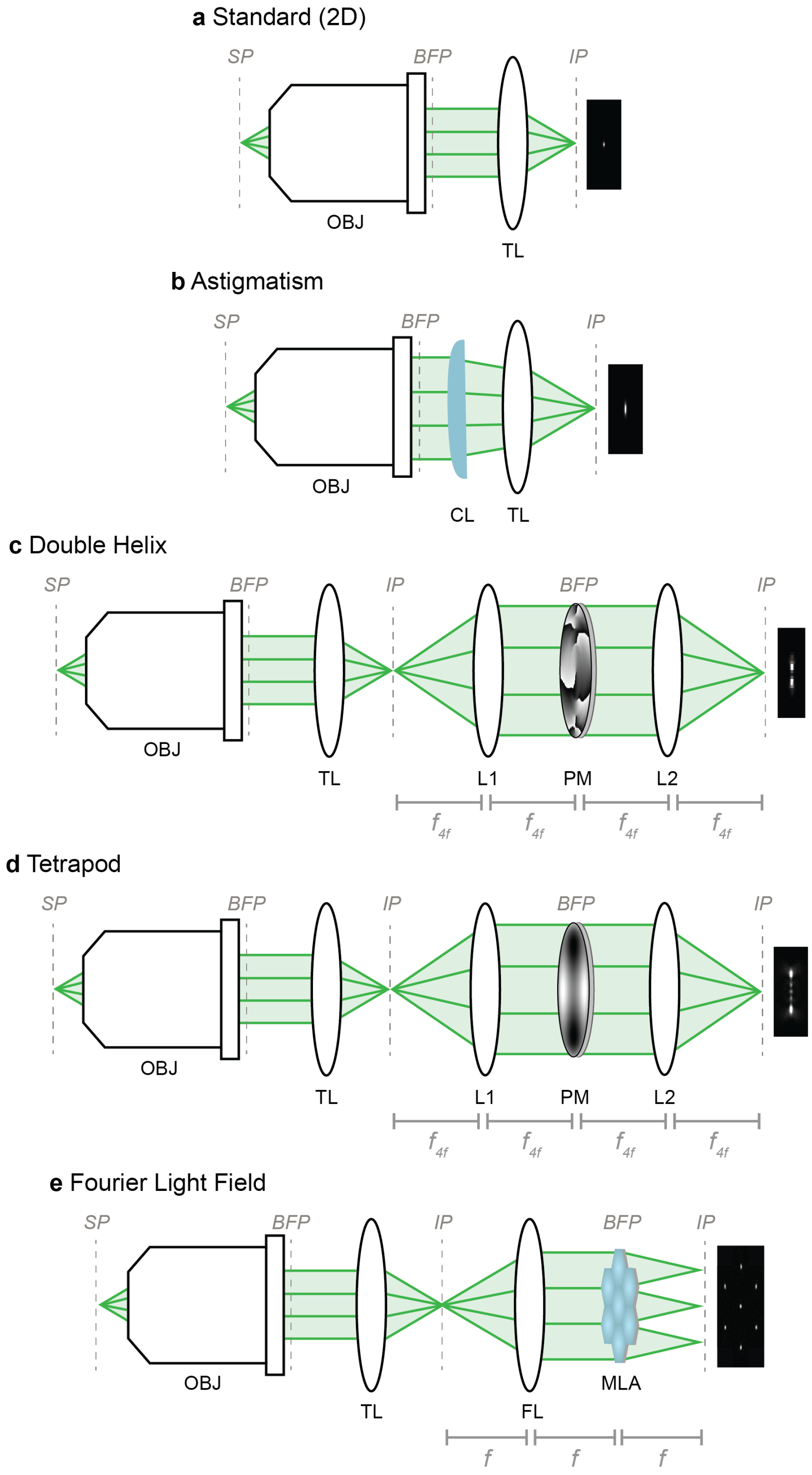

Supplementary Figure 2. Optical configurations to generate each PSF presented in this work. **a** A wide-field microscope in which fluorescence from a point emitter at the sample plane (SP) is collected by an objective (OBJ) and focused onto a detector at the image plane (IP) by the tube lens (TL). The point emitter appears as a diffraction-limited spot. **b** An astigmatism can be induced into the optical PSF by inserting a cylindrical lens (CL) before the detector. **c** Relaying the image plane with a 4f optical configuration of lenses (L1 and L2) grants access to the back focal plane (BFP). Placement of a double helix phase mask in the BFP produces the characteristic double helix pattern for a point-emitter at the conjugate image plane. **d** The tetrapod PSF can be produced in the same way using a tetrapod phase mask. **e** A Fourier light field microscope can be constructed by placing a microlens array in the BFP, which is accessed by use of a fourier lens (FL) in a '3f' configuration.

1.2 SMLFM design parameters

Though consisting of few optical components, a number of design parameters govern the performance of FLFM, a detailed discussion of which can be found in reference [3]. The number of illuminated microlenses along the diameter of the BFP, N, can be determined determined using Eq. (1). This is proportional to the number of perspective views of the sample volume.

$$N = \frac{d_{\text{BFP}}}{P} \tag{1}$$

Where d_{BFP} is the diameter of the BFP and P is the pitch of the MLA. In the present work, N was experimentally measured to be 3.05 by directly imaging the BFP and therefore 3 microlenses fit along the diameter of the BFP. The proportion of the BFP that is segmented by the MLA, also known as *occupancy ratio*, was therefore calculated to be 85%. In SMLFM, depth-of-field (DoF) and precision are limited by the low photon numbers generally yielded by single molecules. Therefore, a hexagonal MLA was implemented here for the first time in a single-molecule regime to improve photon throughput on previous SMLFM iterations that employed square MLAs.

The field-of-view (FoV) must match the spatial scales of the samples being imaged. In theory, the maximum theoretical FoV in SMLFM is that of the objective lens, however for simplicity we define a functional FoV that is the relative size of the central microlens in the BFP. This ensures that microlenses do not overlap. As such, the FoV can be deduced using Eq. (2).

$$\text{FoV} = \frac{P}{M} \qquad M = \frac{f_{\text{tube lens}}}{f_{\text{objective}}} \frac{f_{\text{MLA}}}{f_{\text{Fourier lens}}} \tag{2}$$

Here, M is the magnification of the optical system. The functional FoV obtained in the present work was calculated and measured as a circle of diameter 40 µm.

Finally, the DoF (which scales with the number of illuminated microlenses and hence the diameter of the BFP of the microscope) was determined experimentally to be ~8 µm and calculated at 8.02 µm using Eq. (3).

$$\text{DoF} = 2\lambda \frac{N^2}{n_a^2} + 2 \frac{\delta}{M} \frac{N}{n_a} \tag{3}$$

A summary of these parameters can be found in Supplementary Table 1.

Supplementary Table 1. Summary of the optical parameters of the SMLFM platform described in this study.

Variable	Formula	Value
Objective numerical aperture	n_a	1.27
Objective magnification	M_{obj}	60.0
Objective focal length (mm)	$f_{\text{obj}} = \frac{f_{\text{TL}}}{M_{\text{obj}}}$	3.33
Tube lens focal length (mm)	f_{TL}	200
Fourier lens focal length (mm)	f_{FL}	175
Microlens focal length (mm)	f_{MLA}	175
MLA inradius pitch (mm)	P	2.39
Total magnification	$M = \frac{f_{\text{MLA}}}{f_{\text{obj}}} \cdot \frac{f_{\text{TL}}}{f_{\text{FL}}}$	60.0
BFP diameter (mm)	$d_{\text{BFP}} = 2 \cdot n_a \cdot f_{\text{obj}} \cdot \frac{f_{\text{FL}}}{f_{\text{TL}}}$	7.40
Number of lenses in BFP	$N = \frac{d_{\text{BFP}}}{P}$	3.10
Detector pixel size (µm)	δ	16.0
Field of view (µm)	$\text{FoV} = \frac{P}{M}$	39.8
Depth of field (µm)	$\text{DoF} = 2\lambda \frac{N^2}{n_a^2} + 2 \frac{\delta}{M} \frac{N}{n_a}$	8.2

To confirm that these quantities lead to the full illumination of the MLA, the BFP was located and measured using a camera. The BFP diameter was experimentally measured at 7.3 mm, see Supplementary Fig. 3. Therefore taking the MLA pitch as double the inradius gives an experimental value of $N = 3.05$.

1.3 Improving contrast in SMLFM

An additional functional advantage of SMLFM is that unwanted fluorescence (originating from the solvent, coverslip, excitation beam, etc.) can be spatially localised in the BFP into a single perspective view (see Supplementary Fig. 4).

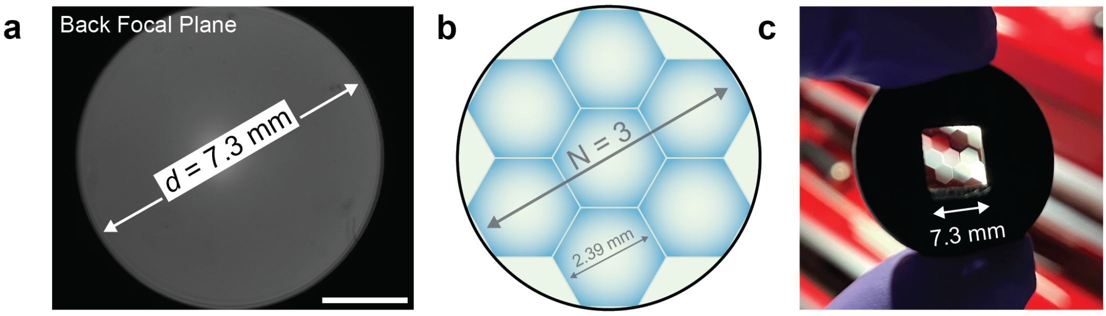

Supplementary Figure 3. Experimentally imaging the BFP. a The BFP (diameter of 7.3 mm) was imaged and measured using flourescein in water as a sample. Scale bar is 2 mm. **b** Superimposition of the MLA on to the BFP, illustrating full illumination of the MLA, corresponding to $N = 3.05$. **c** Photograph of the MLA held in a retaining ring. The MLA is then positioned in an xyz transitional stage in the emission path to ensure fine day-to-day optical alignment.

This cannot be achieved with other 3D modalities examined here as it is a consequence of segmenting the BFP with discrete microlenses that offer spatially defined zones where background fluorescence is collected. We find that the contrast enhancement affords higher localization precision because of a sharp increase in signal-to-noise ratio, despite globally limiting the maximum number of views available for 3D localization.

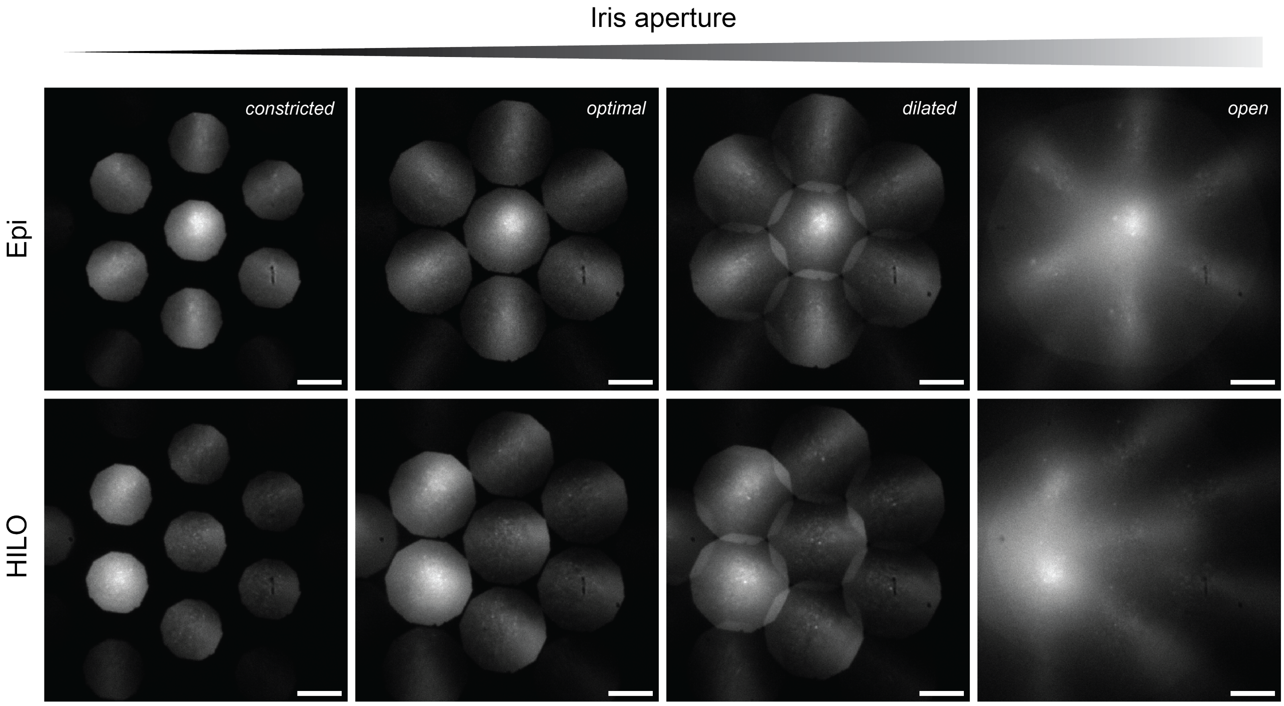

Supplementary Figure 4. Fluorescence background in SMLFM. Fluorescence background from a fluorescent coverslip illuminated under both epifluorescence (Epi) and a highly inclined and laminated optical sheet (HILO), n = 5 fields-of-view. An iris, placed at the conjugate image plane, is used to aperture the imaging area and is presented at increasing dilation. The optimal aperture size maximises field-of-view while avoiding overlapping signals from each microlens. Scale bar is 20 µm.

Supplementary Note 2: Realistic 3D-SMLM Simulations

2.1 Engineered PSF simulations

Simulations of single-molecule images using the standard, astigmatic, double helix and tetrapod PSF were generated using a scaled Fourier transform of the electric field in the back focal plane of the objective due to an isotropic emitter at position $\mathbf{r} = (x_i, y_i, z_i)$. The electric field at the back focal plane of the objective is given by

$$\mathbf{E}_{\text{bfp}}(\rho, \phi; \mathbf{r}) = A(\rho) \exp\left(i\Phi_{\text{xyz}}(\rho, \phi; \mathbf{r})\right) \tag{4}$$

where $A(\rho) = 1/\left(1 - (\text{NA}\rho/n)^2\right)^{1/4}$ is an apodization factor for aplanatic collimation [4], NA is the numerical aperture of the objective, n is the refractive index of the immersion and sample medium, (ρ, ϕ) are normalised polar coordinates in the back focal plane (ρ is normalised to be 1 at the edge of the pupil), and the phase of the electric field is dependent on the position of the emitter in space [5]:

$$\Phi_{\text{xyz}}(\rho, \phi; \mathbf{r}) = k_0 \text{NA}\left(x_i \rho \cos\phi + y_i \rho \sin\phi + z_i \sqrt{\frac{n}{\text{NA}} - \rho^2}\right) \tag{5}$$

The electric field at the image plane is calculated as the scaled Fourier transform of the electric field in the back focal plane:

$$\mathbf{E}_{\text{img}} = \mathcal{F}\left\{\mathbf{E}_{\text{bfp}}(\rho, \phi; \mathbf{r}) \exp\left(i\Phi_{\text{mask}}(\rho, \phi)\right)\right\} \tag{6}$$

where Φ_{mask} is the phase modulation due to a phase mask. The phase masks for the astigmatic and tetrapod PSFs were calculated respectively as scaled primary vertical astigmatism, and a linear combination of primary and secondary vertical astigmatism:

$$\begin{aligned} \Phi_{\text{mask,astig}} &= \frac{2\pi}{5}\rho^2 \cos(2\phi) \\ \Phi_{\text{mask,tetrapod}} &= \frac{\pi}{2}\rho^2 \cos(2\phi) \; - \frac{13\pi}{20}(4\rho^4 - 3\rho^2)\cos(2\phi) \end{aligned}$$

The phase mask for the double helix PSF was calculated using vectorial phase retrieval using an experimental calibration z-scan of fluorescent beads with a diameter of 100 nm [6].
The intensity distribution at the image plane is then calculated from the electric field as:

$$I = \mathbf{E}_{\text{img}}^{\dagger} \mathbf{E}_{\text{img}} \tag{7}$$

where † is the adjoint operator.

2.2 Light field simulations

For single-molecule light field simulations, the electric field in the back focal plane of the objective due to an isotropic emitter is described by Eq. (4). The image formation by a microlens array is modeled as described in reference [7]. The phase of the electric field is modulated by a hexagonal microlens array, and then Fresnel propagated over a distance equal to the focal length of the microlens array [8] as follows:

$$\mathbf{E}_{\text{img}} = \mathcal{F}^{-1}\left\{\mathcal{F}\left\{\mathbf{E}_{\text{bfp}} \exp\left(i\Phi_{\text{MLA}}\right)\right\} \times \exp\left[i\pi\lambda f_{\text{MLA}} \sqrt{f_x^2 + f_y^2}\right]\right\} \tag{8}$$

where the exponential term is the Fresnel transfer function, f_x and f_y are the spatial frequencies in the image plane, and $\mathcal{F}\{\}$ and $\mathcal{F}^{-1}\{\}$ represent the Fourier and inverse Fourier transform respectively. The phase modulation due to the microlens array is given by:

$$\Phi_{\text{MLA}}(\rho, \phi) = \left[\text{hex}\left(x_{uv}, y_{uv}\right) \exp\left(\frac{-ik}{2f_{\text{MLA}}}(x_{uv}^2 + y_{uv}^2)\right)\right] * \text{comb}(\rho, \phi) \tag{9}$$

where the term between square brackets describes the phase of a single hexagonal microlens and (x_{uv}, y_{uv}) are normalised coordinates in a single microlens. The first term between the square brackets is a hexagonal binary amplitude mask, and the exponential term is the phase transformation due to a thin lens [9] with a focal length f_{MLA}. The phase

modulation induced by the entire MLA is obtained by tiling the phase modulation due to a single microlens in a hexagonal pattern through convolution with a hexagonal 2D comb function that specifies the position of the centers of the microlenses. The intensity at the image plane is calculated from the electric field in the image plane using Eq. (7).

2.3 Detection noise model

Detection by an EMCCD camera is modelled as in [10]

$$n_{ie}(x,y) = \mathcal{P}\{\mathrm{QE} \cdot [I(x,y) + b] + c\} \tag{10}$$

$$I_{\text{camera}}(x,y) = \Gamma\{n_{ie}(x,y), g_{em}\} + \mathcal{G}(0, \sigma_{rn}) + \mathcal{O} \tag{11}$$

where $I(x,y)$ is the intensity distribution at the image plane in photons, b is a constant fluorescence background in photons, $n_{ie}(x,y)$ is the number of input electrons, $\mathcal{P}$ represents a Poisson distribution, QE is the quantum efficiency of the camera, c is the spurious charge, Γ represents a gamma function, g_{em} is the electron multiplication gain, $\mathcal{G}$ represents a Gaussian distribution with a zero mean and standard deviation σ_{rn}, and $\mathcal{O}$ is a constant offset. Detector saturation is taken into account.

2.4 Simulation parameters

Simulations were performed for standard, astigmatic, double helix, tetrapod and light field PSF at different emitter densities (between N= 2 and N= 150 emitters/volume), with three times 100 independent repeats for each density. A fixed number of emitters N were randomly placed in a 3D volume with a specific DoF and FoV as summarised in Supplementary Table 2. The fluorescence background was fixed across simulations and set to 10 photons per 266 nm pixel. For the light field simulations, the background per pixel was divided by the number of microlenses in the pupil (seven in this case), as the background is also divided into the different perspective views. Simulations were performed for three signal levels: 1,000, 4,000, and 10,000 detected photons/emitter. For each PSF, a z-stack was also simulated of a single bright emitter (10,000 detected signal photons, no background photons) covering the full DoF of the method for calibration.

Supplementary Table 2. Simulation parameters.

PSF	DoF (µm)	FoV (µm^2)	Pixel size (nm)
Standard	0	20×20	110
Astigmatic	1	20×20	110
Double helix	4	20×20	266
Light field	8	20×20	266
Tetrapod	8	20×20	110

The following system parameters were used for the light field simulations: hexagonal microlens array with a pitch of 2.39 mm, microlens focal length of 175 mm, fourier lens focal length of 175 mm, tube lens focal length of 200 mm (Nikon), and a 60× water-immersion objective (Nikon) with NA = 1.27. The camera parameters match the Evolve Delta 512 EMCCD camera from Photometrics, i.e. a physical pixel size of 16 µm.

The following values were used for the detection noise parameters: QE $= 1$ electrons/photon, $c = 0.002$ electrons, $g_{em} = 250$ counts/electron (counts = analog-to-digital unit), $\sigma_{rn} = 74.4$ electrons, $\mathcal{O} = 400$ counts. These parameters correspond to the EMCCD camera Evolve Delta 512 from Photometrics.

Supplementary Note 3: Cramér–Rao Lower Bound

The Cramér-Rao lower bound (CRLB) is the theoretical limit of the variance of an unbiased estimator, in this case the 3D localization of a molecule. Assuming Poisson distributed noise, the CRLB can be determined by the inverse of the Fisher information matrix, as described in [11] and Eq. (12).

$$F_x = \sum_{k=1}^{N} \frac{1}{S(k)+b} \left(\frac{\partial S(k)}{\partial x} \right)^2 \tag{12}$$

Where N is the total number of pixels on the sensor, $S(k)$ is the signal for the kth pixel and b is the background signal. The signal, $S(k)$, is calculated as the PSF modeled as described in Supplementary Note 2.2 using parameters that match the experimental setup detailed in Methods.

The hexagonal SMLFM platform described in the present work was simulated for 4,000 and 50,000 detected photons to determine the maximum theoretical precision of the instrument for typical experimental photon values and an unlimited photon scenario. The CRLB was computed for these images over the whole axial range (8 µm) and presented in Supplementary Fig. 5.

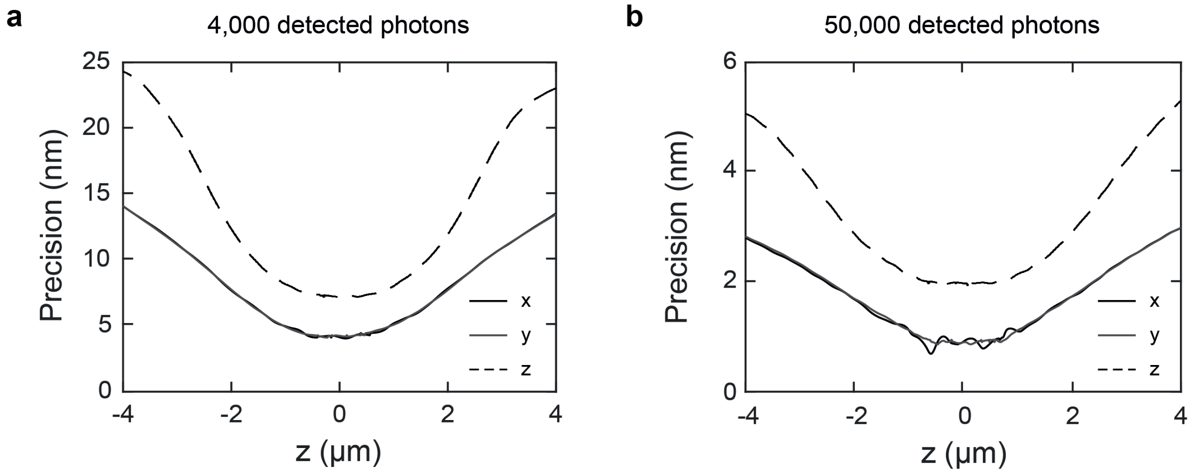

Supplementary Figure 5. Lateral and axial Cramér–Rao lower bounds. Lateral and axial Cramér–Rao lower bounds for the experimental setup discussed in this work determined through simulations for **a** 4,000 detected photons and **b** 50,000 detected photons with 150 background photons.

The precision of the optical setup at typical single-molecule photon values was determined to be below 14 nm laterally and 24.5 nm axially over the whole 8 µm DoF. Precision reaches a maximum of 5 nm laterally and 8 nm axially at the middle of the focal volume for 4,000 photons. This improves to $\leq$2 nm in the photon-unlimited case. Although the lateral and axial precision is not isotropic across the whole DoF these results are consistent with previous work [1] that employed a square MLA and exhibits improved localization precision throughout.

Supplementary Note 4: Analysis of Simulated Data

4.1 Defining density

In this study we define *density of emitters* as the number of single emitters that give rise to an intensity distribution on a 2D detector per frame (per unit area). This approach aligns with previous work [12] and is consistent with the projection of a 3D volume onto a 2D array of pixels. A consistent 20×20 µm FoV meant that emitter densities spanned the range 0.005 to 0.375 μm^{-2} with 0.100 μm^{-2} resembling a typical labelling density for 2D-SMLM experiments [13]. Supplementary Fig. 6 shows the variation in the PSF size throughout the corresponding DoF established through intensity thresholds.

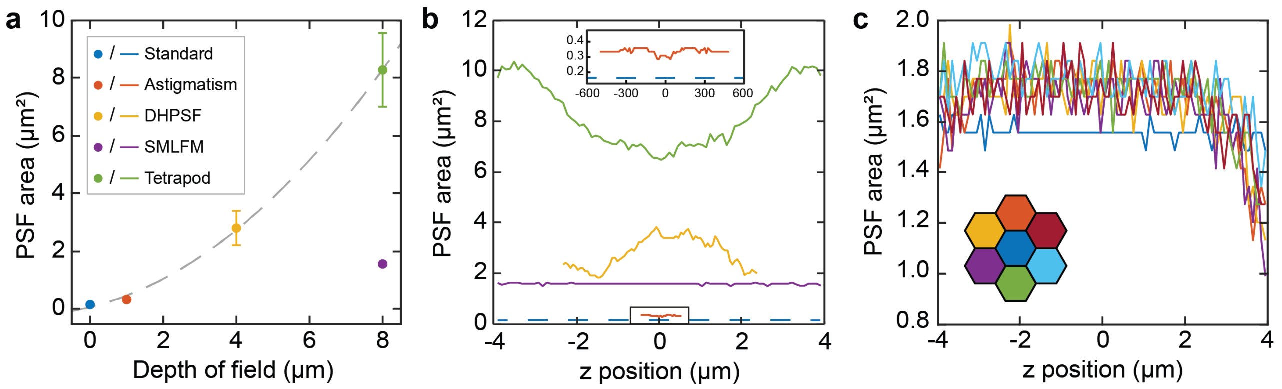

Supplementary Figure 6. The SMLFM PSF is highly compact and size-invariant across the whole DoF compared to other 3D PSFs. a Area of each 3D PSF explored herein as a function of the respective depth-of-field. Data points represent the average PSF size, determined via thresholds, over the respective depth-of-field (n = 80 steps) where error bars represent the 1st standard deviation. The grey dashed line indicates the general trend. SMLFM (purple) significantly deviates from the trend, indicating the PSF is much more compact than expected for the DoF. **b** Area of each 3D PSF across the respective DoF. The SMLFM PSF (central view) is size invariant across the whole DoF, a result of the low effective numerical aperture of each microlens. This enables the PSF to be fitted with a conventional 2D gaussian function across the whole DoF prior to 3D reconstruction. **c** SMLFM PSF size as a function of axial position for each perspective view. Axial size invariance pertains in the outer perspective views over only a marginally larger detector area. All PSF area values were determined by realistic calibration datasets using an intensity-based threshold. Data representative of 3 repeats.

4.2 Optimising pixel size

The optimal magnification of a microscope platform depends on the number of photons (signal) and background noise. In general, the pixel size is equivalent to the standard deviation of the PSF, as described in the well-established work from the group of Watt W. Webb [14].

When simulating the 3D PSF data, we took this into consideration by ensuring that the chosen pixel size yielded optimal performance for quantitative comparison and resembled experimental data. We illustrate the importance of magnification in Supplementary Fig. 7 using the DHPSF as an example. Here, the SNR is significantly degraded at 110 nm, which would result in poorer spot detection and poorer PSF fitting compared to a pixel size of 266 nm. Therefore, a pixel size of 110 nm is implemented for standard (2D), astigmatism, and the tetrapod PSF simulated data, while a 266 nm pixel size was implemented for SMLFM and the DHPSF. Note that pixel size does not substantially affect the maximum achievable density, as the physical region of the detector used for imaging is identical between techniques.

4.3 Defining speed improvement

We quantified the maximum speed difference between SMLFM and the current state-of-the-art DHPSF (with which we possess most experimental experience). When determining this speed enhancement it was important to consider the optimal localization densities for both techniques to ensure a fair comparison. Therefore, we compared the emitter density at which both techniques shared the same error rate (i.e. the value at which the proportion of true positive localizations relative to the ground truth was equal). Plots of error rate vs. density are presented in Supplementary Fig. 8 with the difference between the two interpolated curves (corresponding to the relative speed of each technique) plotted directly above.

Speed improvement was also calculated at different values of detected photons to reflect different labelling scenarios: a fluorescent protein (1,000 photons), an organic dye (4,000 photons) and a next-generation probe (10,000 photons). We

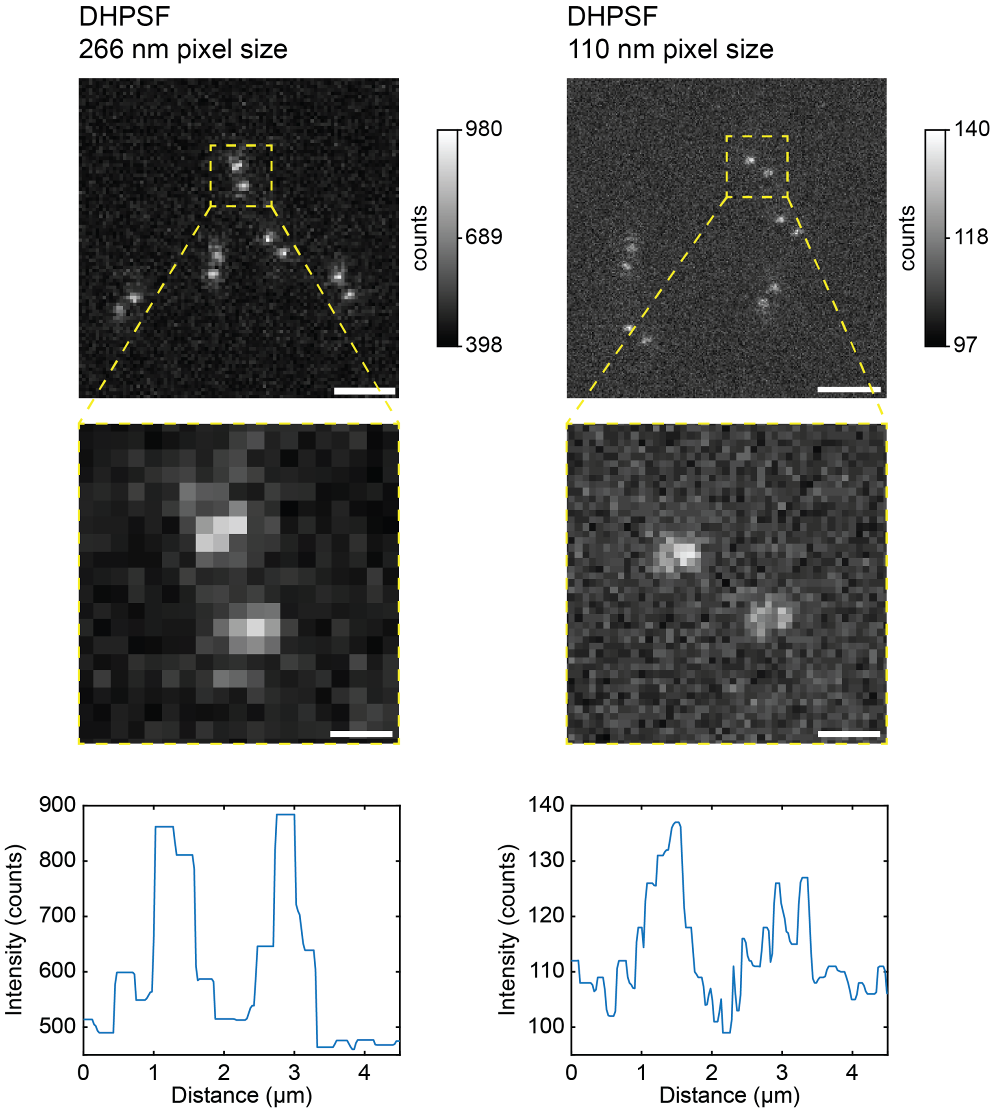

Supplementary Figure 7. The effect of pixel size on SNR and sampling. Optimal pixel size (magnification) has been shown by Thompson et al. to depend on signal-to-noise ratio (SNR) and generally equates to the standard deviation of the PSF [14]. When simulating 3D PSFs, the pixel size was chosen to optimise SNR whilst also ensuring adequate spatial sampling of the respective PSF. This effect is illustrated here using the DHPSF, which was simulated at a pixel size of 266 nm (left) and 110 nm (right). The SNR is significantly degraded at 110 nm compared to 266 nm, which leads to both poor PSF detection and localization precision. Scale bar is 5 μm and 1 μm for the zoomed regions.

determined SMLFM to facilitate a maximum speed improvement of 25.3× (40.0% error rate), 8.95× (13.4% error rate) and 10.6× (13.4% error rate) at 1,000, 4,000 and 10,000 detected photons, respectively.

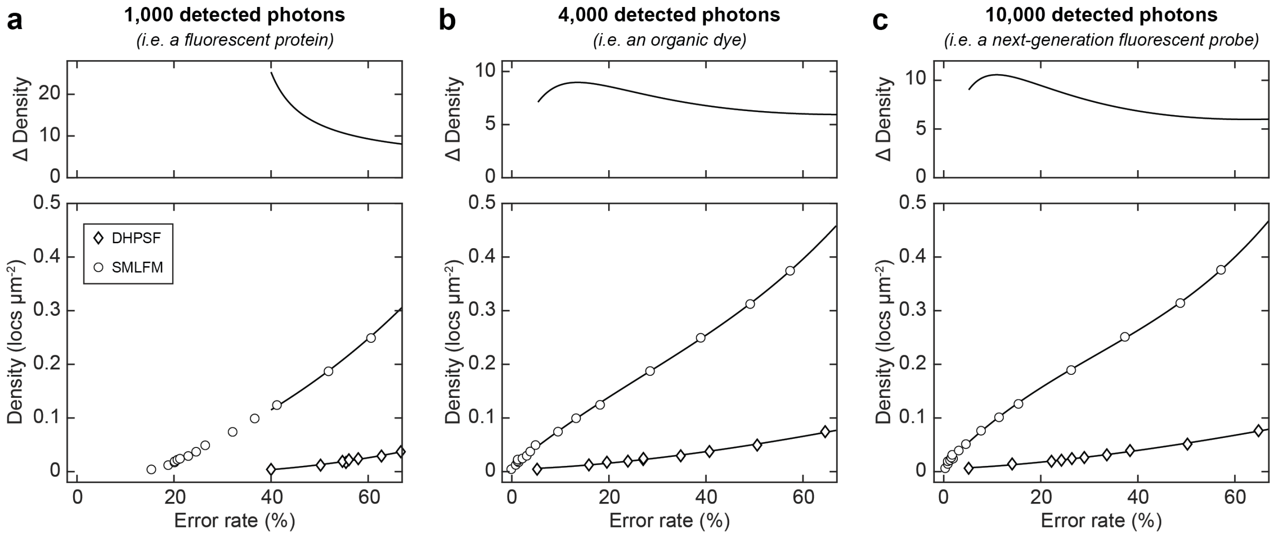

Supplementary Figure 8. Relative speed of SMLFM compared to the DHPSF for typical photon values. The density of single emitters per frame for a given error rate (proportion of false negatives relative to the number of ground truth localizations) for SMLFM and DHPSF simulated at **a** 1,000 detected photons, **b** 4,000 detected photons and **c** 10,000 detected photons. Plots of the density difference between the two interpolated lines (relative speed) are presented above each graph. To avoid extrapolation, at 1,000 photons a density difference is only calculated after an error rate of 40%. Data representative of 3 repeats.

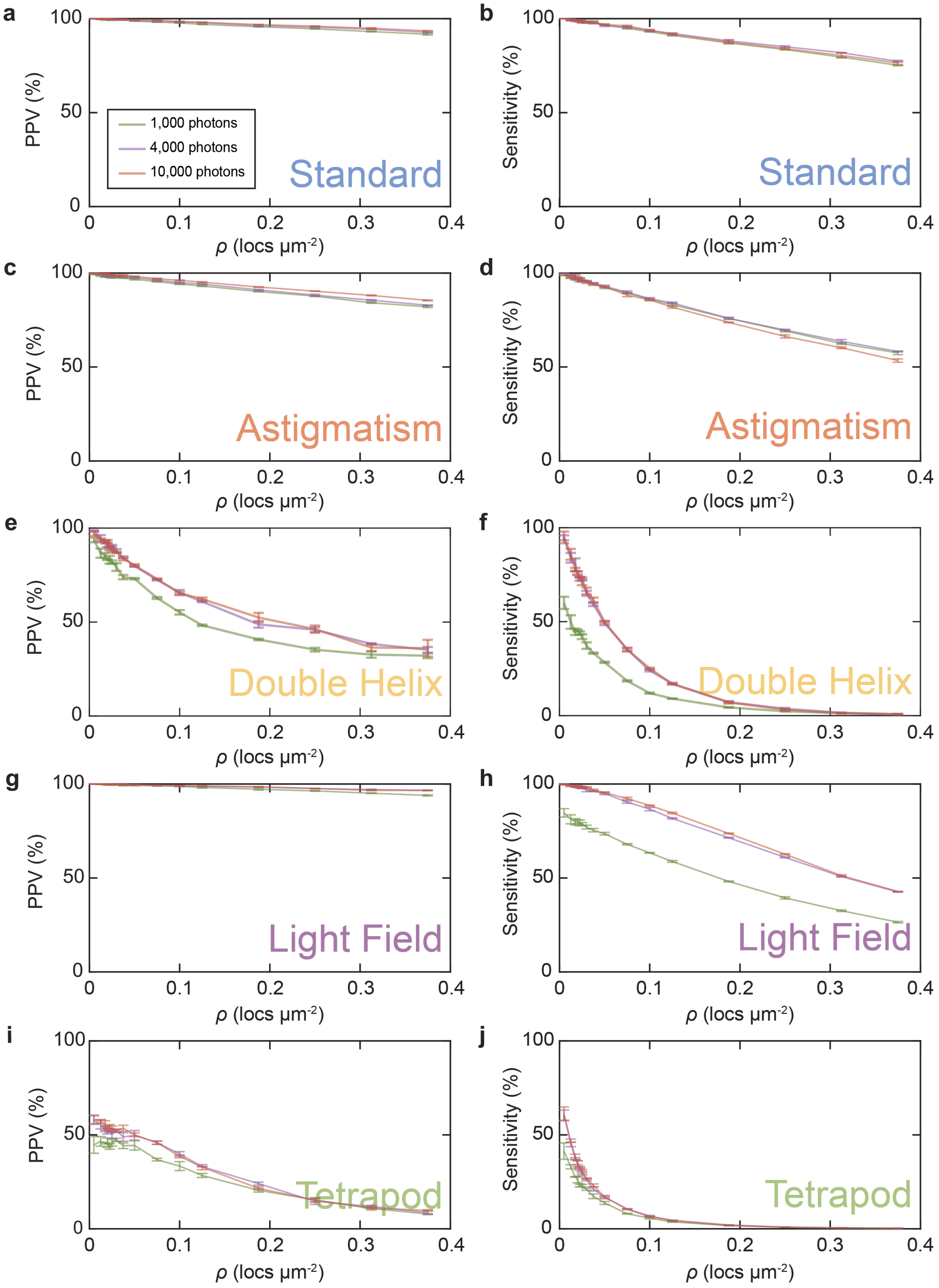

Supplementary Figure 9. Positive predictive value (PPV) and sensitivity at different signal-to-noise ratios. Graphs of PPV and sensitivity against emitter density for the standard (**a** and **b**), astigmatism (**c** and **d**), double helix (**e** and **f**), light field (**g** and **h**) and tetrapod (**i** and **j**) PSFs simulated for 1,000, 4,000 and 10,000 detected photons (n = 3 repeats of 100 frames). Error bars represent the 1st standard deviation from the mean.

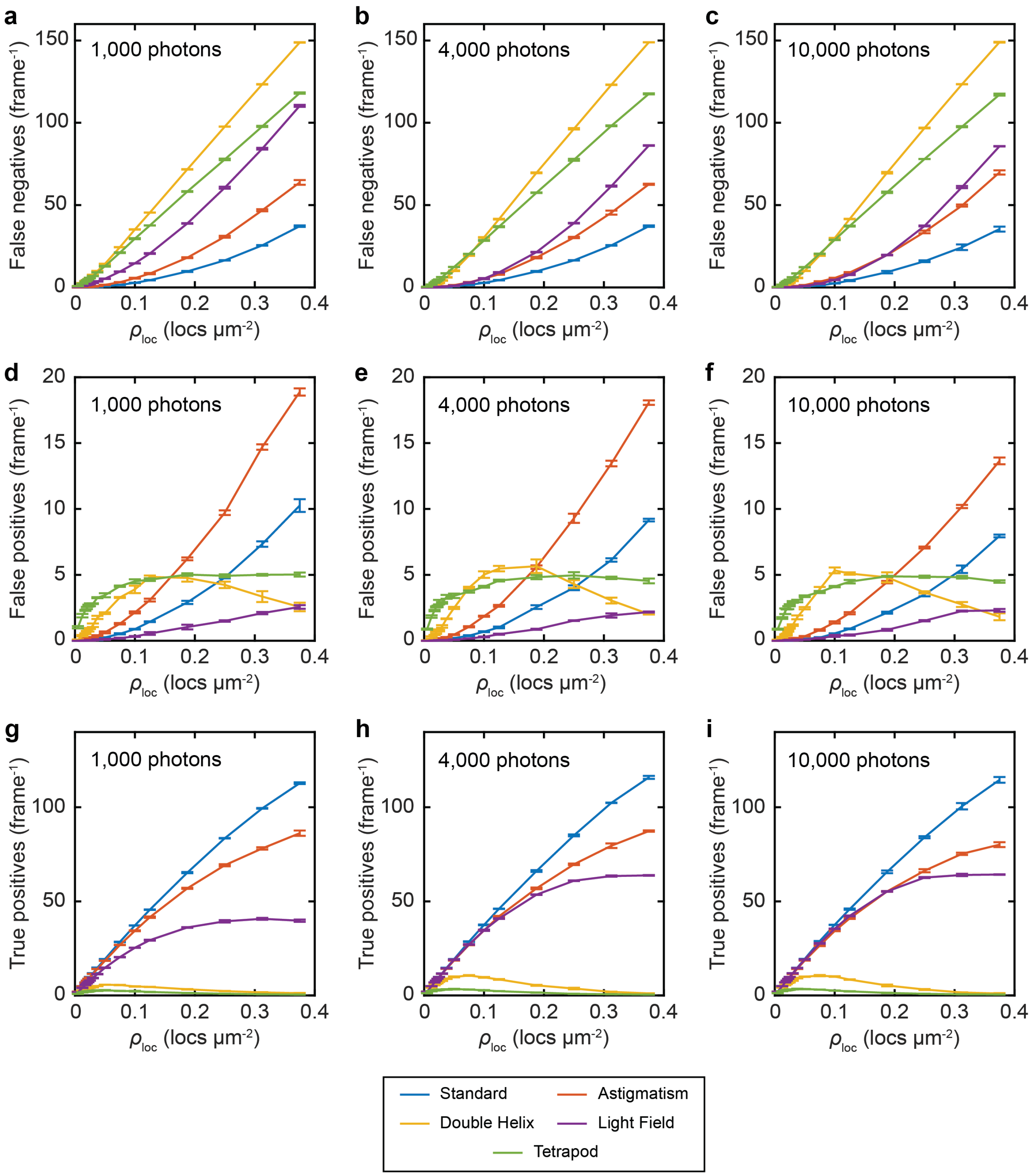

Supplementary Figure 10. Imaging metrics from the analysis of simulated localization datasets. False negative localizations per frame as a function of emitter density at **a** 1,000, **b** 4,000, and **c** 10,000 detected photons determined through simulations described in Supplementary Note 2. False positive localizations per frame as a function of emitter density at **d** 1,000, **e** 4,000, and **f** 10,000 detected photons. True positive localizations per frame as a function of emitter density at **g** 1,000, **h** 4,000, and **i** 10,000 detected photons. Each plot represents the average value (n = 3 repeats of 100 frames) where error bars represent the 1st standard deviation from the mean. A 90° rotation filter was applied to each frame during the matching analysis to account for the higher likelihood of incorrectly pairing a fitted point with a ground truth coordinate at high localization densities.

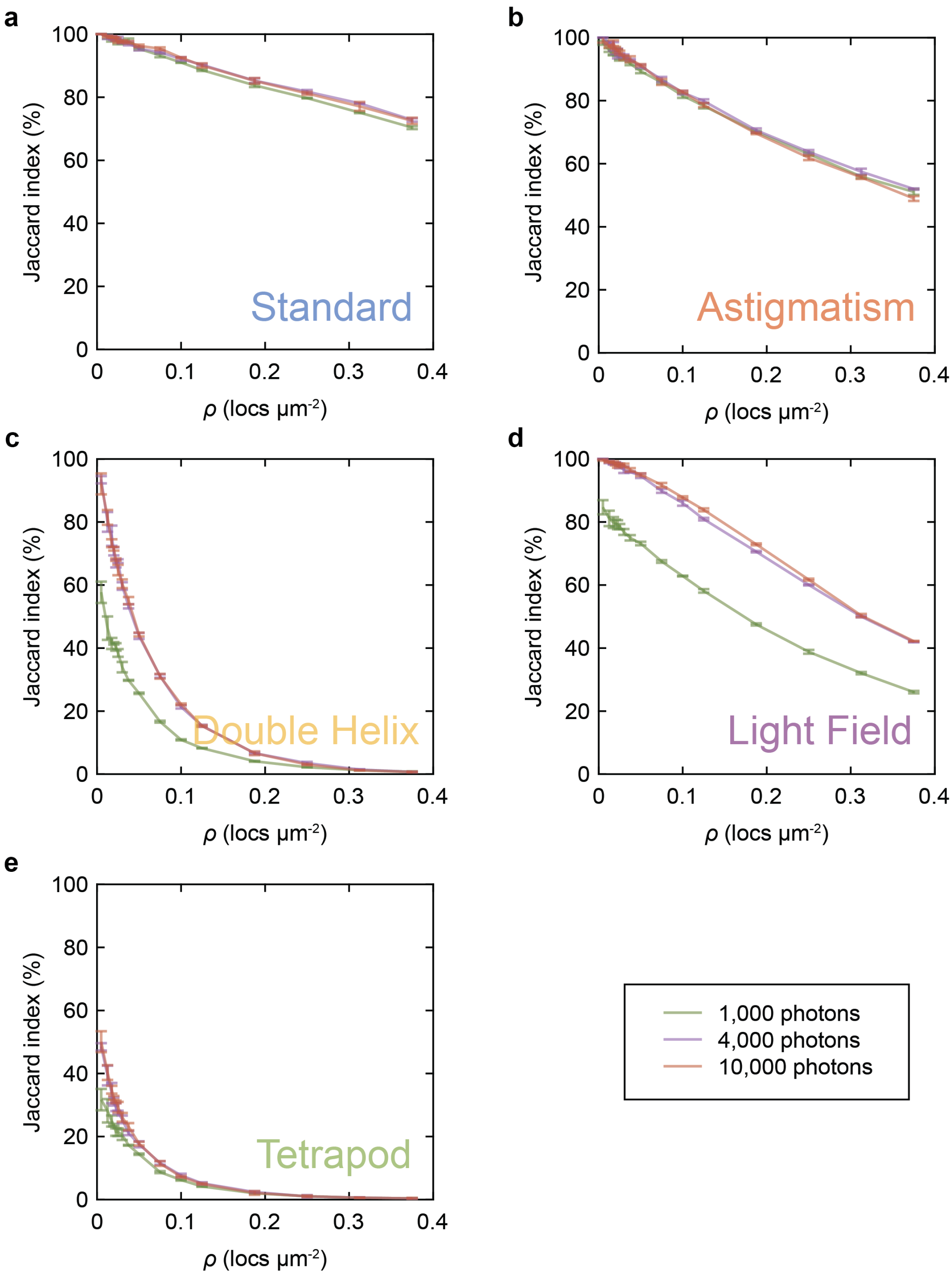

Supplementary Figure 11. Comparison of Jaccard index as a function of emitter density. Jaccard index is a commonly used metric to describe the performance of SMLM techniques. Described as $TP\ /\ TP + FP + FN$, Jaccard index is presented here as a function of emitter density for the **a** standard, **b** astigmatic, **c** double helix, **d** light field, and **e** tetrapod PSFs (n = 3 repeats of 100 frames). Error bars represent the 1st standard deviation from the mean.

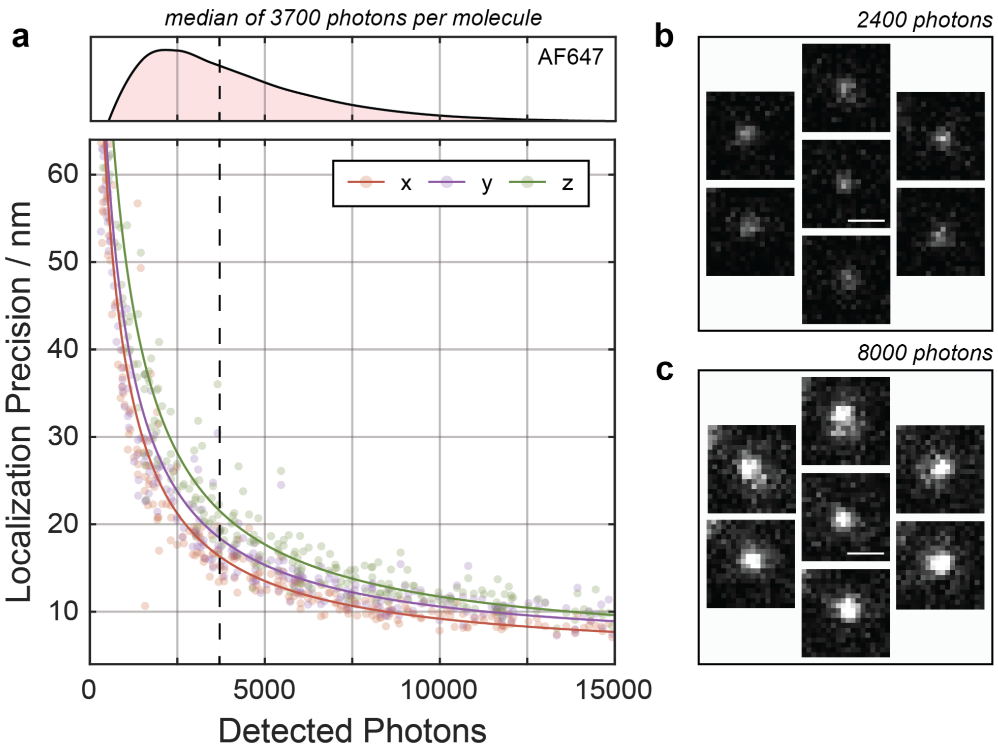

Supplementary Figure 12. Localization precision of SMLFM. a The standard deviation in the lateral (x,y) and axial (z) position of 100 nm TetraSpeck microspheres was measured at different fluorescent intensities using SMLFM. For each field-of-view, 200 frames were accumulated with an exposure time of 30 ms. Histogram of detected photons for each molecule of AF647 is presented above, illustrating a precision of ~18 nm laterally and ~23 nm axially for BCR and tubulin imaging. Example fluorescent intensities captured through SMLFM are shown on the right for **b** 2,400 and **c** 8,000 detected photons. These results demonstrate that SMLFM affords greater isotropy in localization precision when imaging biological molecules in cells compared to the DHPSF [15]. Scale bar is 1 µm.

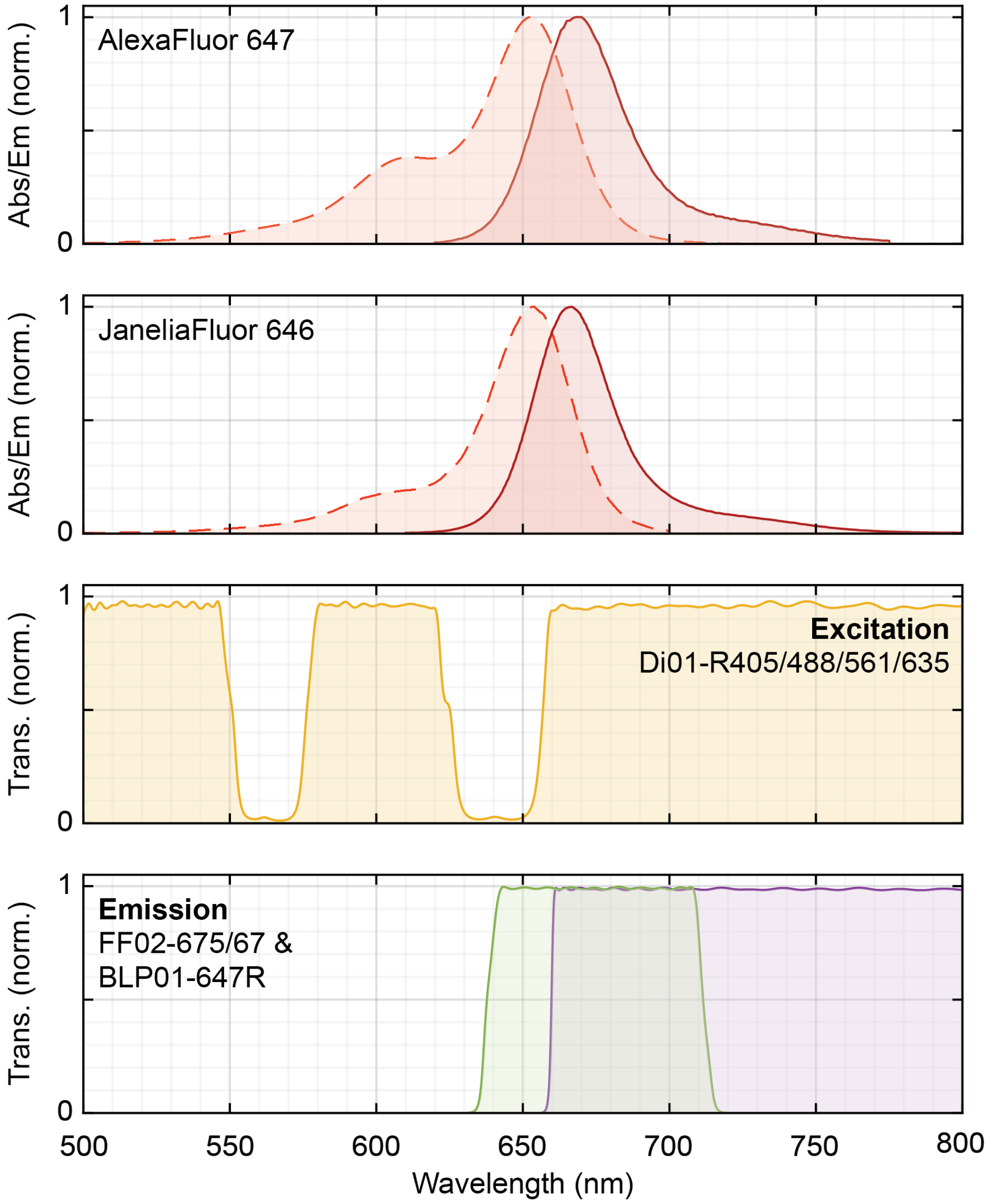

Supplementary Figure 13. Optical properties of fluorescent probes and filters. Normalised absorbance (dashed line) and emission (solid line) spectra for AF647 and JF646 used in this study. Excitation and emission filters are presented as a function of normalised transmission.

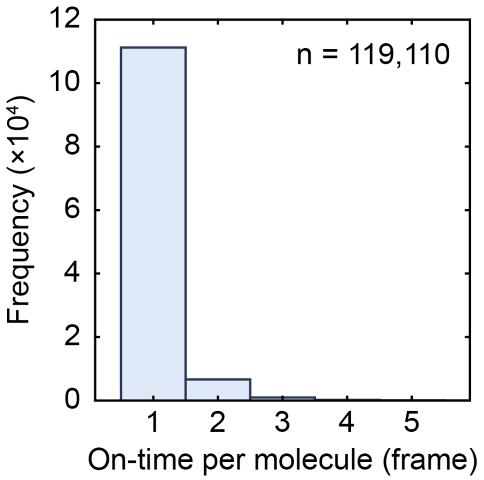

Supplementary Figure 14. Photoswitching kinetics of AF647 in dSTORM experiments. The optimisation of AF647 photoswitching kinetics in dSTORM experiments meant that 93.5% of all fluorescent events were captured in a single frame. However, temporal grouping was applied to all dSTORM data to ensure that the 5.6% and 0.8% of fluorescent events occurring over two and three frames, respectively, were attributed to a single molecule of AF647. As such, an upper bound of 4 frames was taken for temporal grouping, which led to a ~5% reduction in the number of localizations per dataset (n = 119,110 localizations in total).

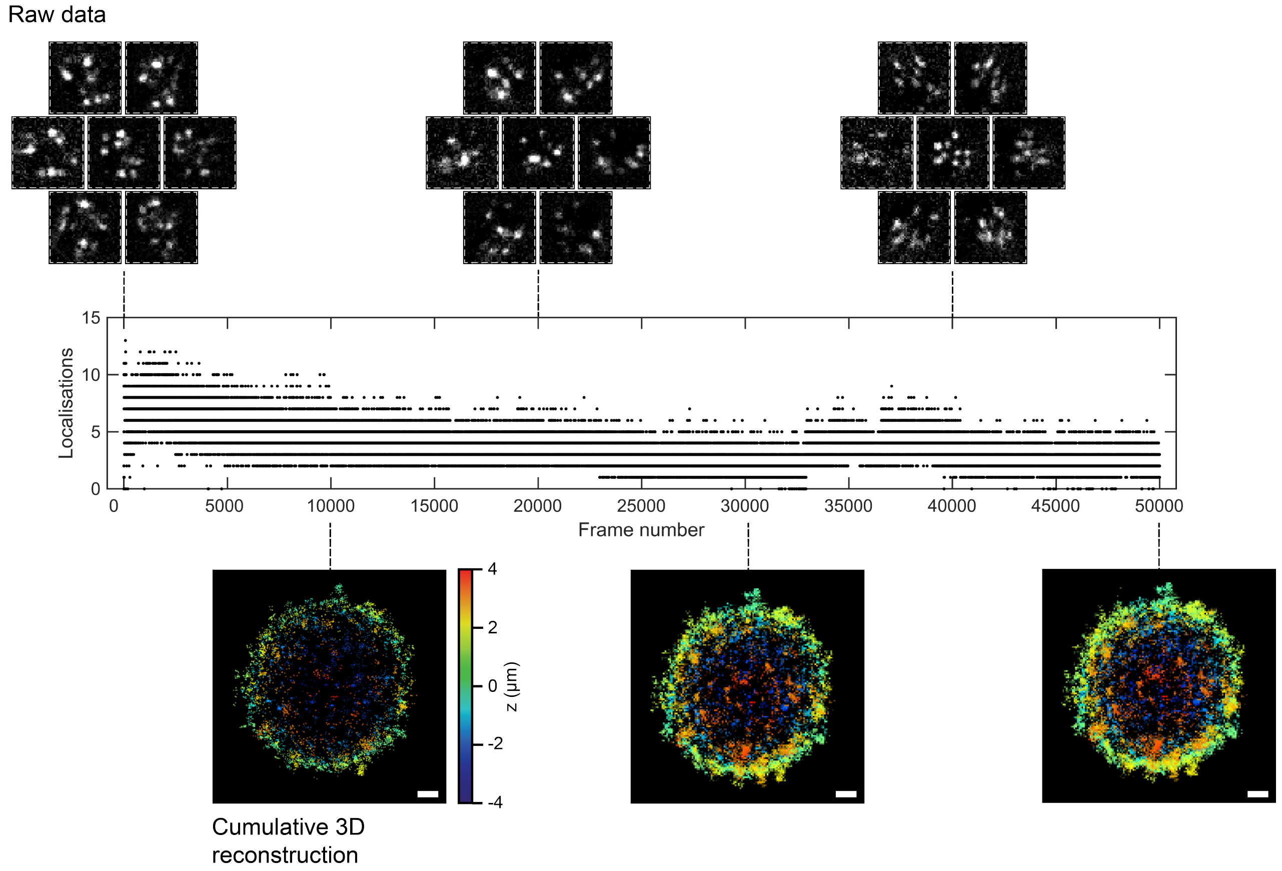

Supplementary Figure 15. Localization rate during dSTORM imaging of the BCR. Single cropped frames of raw data is presented on top and the cumulative 3D reconstruction of localizations per frame shown below. An additional laser at 405 nm was used commencing at frame 33,000 and increased in power at frame 36,000 to ensure all fluorophores had been imaged. Scale bar is 1 µm. Data representative of 3 repeats.

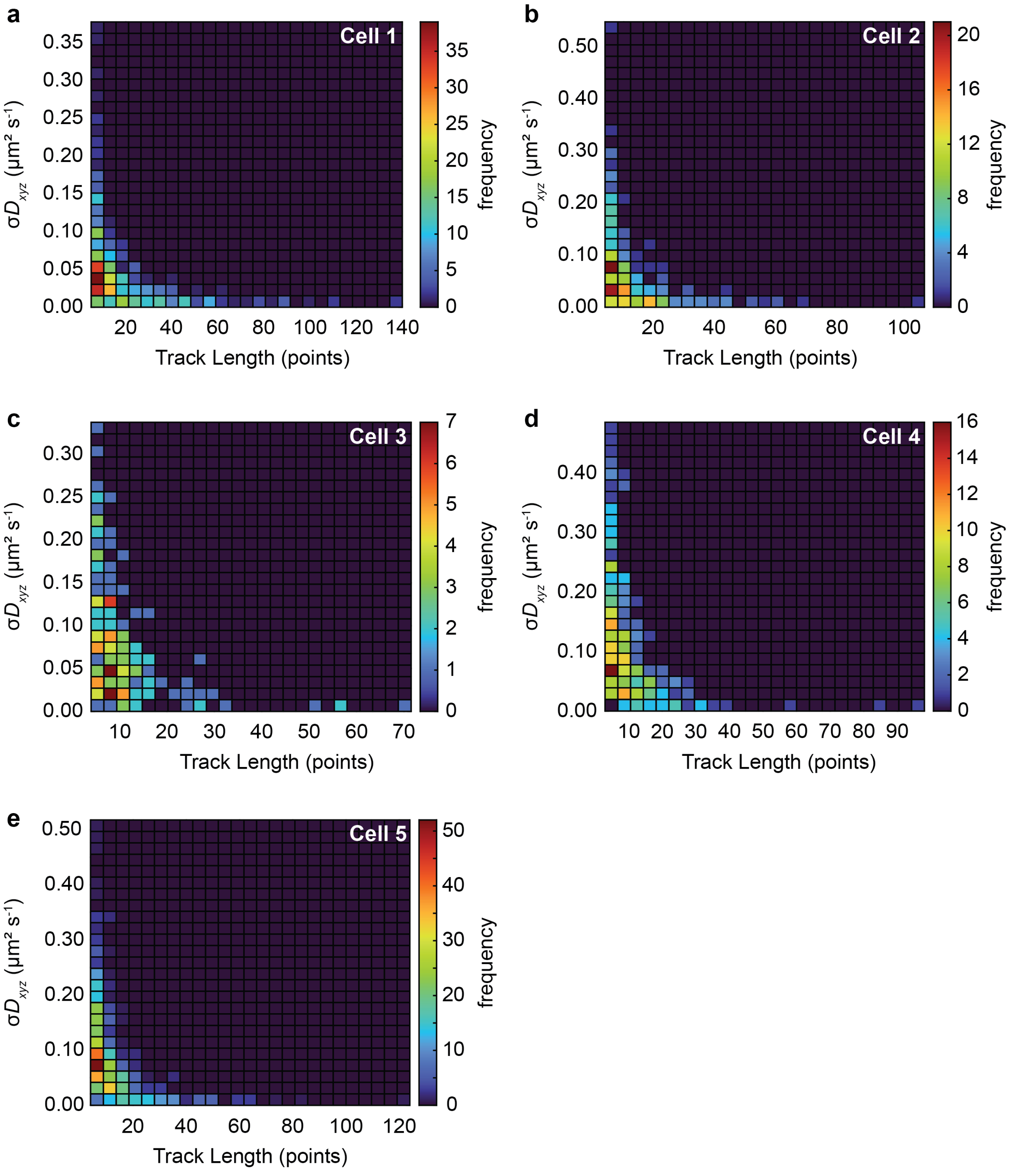

Supplementary Figure 16. Maximum likelihood error for diffusion measurements. 2D histograms showing the maximum likelihood error for diffusion measurements in xyz as a function of track length for each cell (**a**–**e**) that contributes to the data presented in Fig. 4.

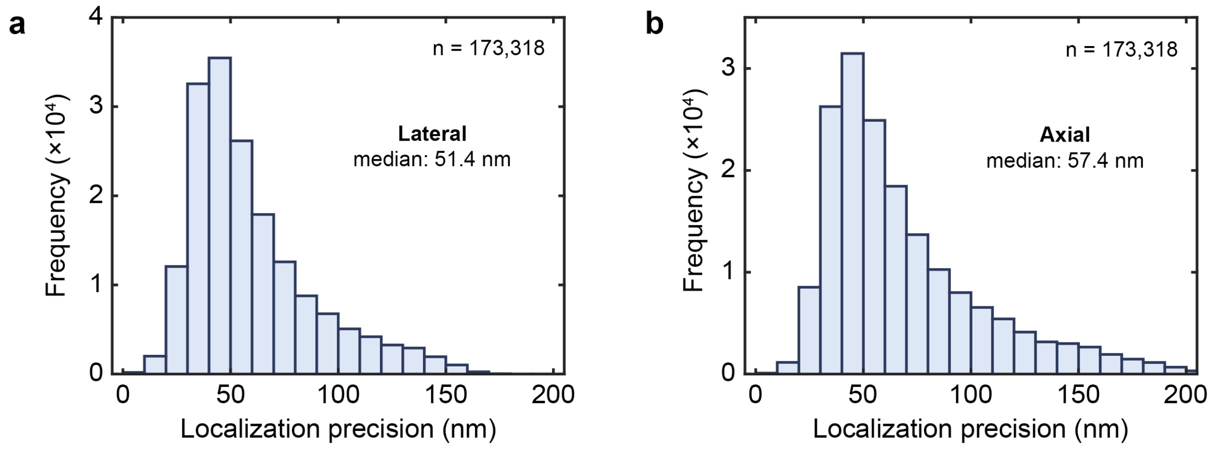

Supplementary Figure 17. Lateral and axial localization precision in tubulin image. Localization precision obtained **a** laterally and **b** axially in the high-density tubulin image presented in Fig. 5.

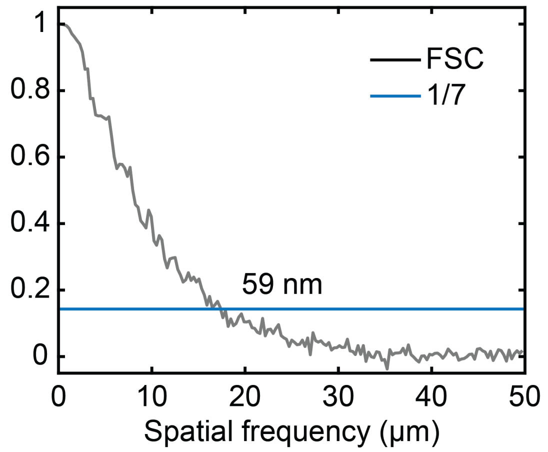

Supplementary Figure 18. Resolution of high-density tubulin dataset. Fourier shell correlation (FSC) estimates an isotropic resolution of 59 nm at a 1/7 resolution criteria for the high-density tubulin image presented in Fig. 5. Data representative of 3 repeats.

Supplementary References

1. Ruth R. Sims, Sohaib Abdul Rehman, Martin O. Lenz, Sarah I. Benaissa, Ezra Bruggeman, Adam Clark, Edward W. Sanders, Aleks Ponjavic, Leila Muresan, Steven F. Lee, and Kevin O'Holleran. Single molecule light field microscopy. *Optica*, 7(9):1065, September 2020.
2. Changliang Guo, Changliang Guo, Wenhao Liu, Wenhao Liu, Xuanwen Hua, Haoyu Li, and Shu Jia. Fourier light-field microscopy. *Optics Express*, 27(18):25573–25594, September 2019. Publisher: Optical Society of America.
3. Laura Galdón, Genaro Saavedra, Jorge Garcia-Sucerquia, Manuel Martínez-Corral, and Emilio Sánchez-Ortiga. Fourier lightfield microscopy: a practical design guide. *Applied Optics*, 61(10):2558, March 2022.
4. J. Kim, Y. Wang, and X. Zhang. Calculation of vectorial diffraction in optical systems. *JOSA A*, 35:526–535, 2018.
5. E.J. Botcherby, R. Juskaitis, M.J. Booth, and T. Wilson. An optical technique for remote focusing in microscopy. *Optics Communications*, 281:880–887, 2008.
6. Boris Ferdman, Elias Nehme, Lucien E. Weiss, Reut Orange, Onit Alalouf, and Yoav Shechtman. VIPR: vectorial implementation of phase retrieval for fast and accurate microscopic pixel-wise pupil estimation. *Optics Express*, 28(7):10179–10198, March 2020. Publisher: Optica Publishing Group.
7. C. Guo, W. Liu, X. Hua, H. Li, and S. Jia. Fourier light-field microscopy. *Optics Express*, 27(18):25573–25594, 2019.
8. D.G. Voelz. *Computational fourier optics : a MATLAB tutorial*. SPIE, 1st edition, 2011.
9. J. Goodman. *Introduction to Fourier Optics*. W.H.Freeman, Co Ltd, 3rd edition, 2005.
10. Daniel Sage, Thanh-An Pham, Hazen Babcock, Tomas Lukes, Thomas Pengo, Jerry Chao, Ramraj Velmurugan, Alex Herbert, Anurag Agrawal, Silvia Colabrese, Ann Wheeler, Anna Archetti, Bernd Rieger, Raimund Ober, Guy M. Hagen, Jean-Baptiste Sibarita, Jonas Ries, Ricardo Henriques, Michael Unser, and Seamus Holden. Super-resolution fight club: assessment of 2D and 3D single-molecule localization microscopy software. *Nature Methods*, 16(5):387–395, May 2019.
11. Raimund J. Ober, Sripad Ram, and E. Sally Ward. Localization accuracy in single-molecule microscopy. *Biophysical Journal*, 86(2):1185–1200, February 2004.
12. Elias Nehme, Daniel Freedman, Racheli Gordon, Boris Ferdman, Lucien E. Weiss, Onit Alalouf, Tal Naor, Reut Orange, Tomer Michaeli, and Yoav Shechtman. DeepSTORM3d: dense 3d localization microscopy and PSF design by deep learning. *Nature Methods*, 17(7):734–740, June 2020.
13. Sebastian van de Linde, Anna Löschberger, Teresa Klein, Meike Heidbreder, Steve Wolter, Mike Heilemann, and Markus Sauer. Direct stochastic optical reconstruction microscopy with standard fluorescent probes. *Nature Protocols*, 6(7):991–1009, June 2011.
14. Russell E Thompson, Daniel R Larson, and Watt W Webb. Precise nanometer localization analysis for individual fluorescent probes. *Biophysical Journal*, 82(5):2775–2783, May 2002.
15. Alexander R. Carr, Aleks Ponjavic, Srinjan Basu, James McColl, Ana Mafalda Santos, Simon Davis, Ernest D. Laue, David Klenerman, and Steven F. Lee. Three-Dimensional Super-Resolution in Eukaryotic Cells Using the Double-Helix Point Spread Function. *Biophysical Journal*, 112(7):1444–1454, April 2017.
