# Supplementary material for: High-density volumetric super-resolution microscopy
Source: Nat Commun. 2024 Mar 2;15:1940. doi: 10.1038/s41467-024-45828-5 (PMC10908787; doi:10.1038/s41467-024-45828-5)
Supplement: Supplementary file 3 — Description of Additional Supplementary Files [file 41467_2024_45828_MOESM3_ESM.pdf]

**Description of Additional Supplementary Files**

File Name: Supplementary Movie 1

Description: Simulated 3D PSF datasets at increasing emitter densities ($\rho_{loc}$).

File Name: Supplementary Movie 2

Description: Comparison of simulated and experimental SMLFM data.

File Name: Supplementary Movie 3

Description: B-cell raw localization data and 3D reconstruction.

File Name: Supplementary Movie 4

Description: 3D reconstruction of BCR diffusion across a live cell membrane.
