# Supplementary material for: High-density volumetric super-resolution microscopy
Source: Nat Commun. 2024 Mar 2;15:1940. doi: 10.1038/s41467-024-45828-5 (PMC10908787; doi:10.1038/s41467-024-45828-5)
Supplement: Supplementary file 8 — Reporting Summary [file 41467_2024_45828_MOESM8_ESM.pdf]

nature portfolio

Corresponding author(s): Steven F. Lee

Last updated by author(s): Jan 9, 2024

Reporting Summary

Nature Portfolio wishes to improve the reproducibility of the work that we publish. This form provides structure for consistency and transparency in reporting. For further information on Nature Portfolio policies, see our Editorial Policies and the Editorial Policy Checklist.

Statistics

For all statistical analyses, confirm that the following items are present in the figure legend, table legend, main text, or Methods section.

n/a	Confirmed	
[ ]	[x]	The exact sample size (n) for each experimental group/condition, given as a discrete number and unit of measurement
[ ]	[x]	A statement on whether measurements were taken from distinct samples or whether the same sample was measured repeatedly
[x]	[ ]	The statistical test(s) used AND whether they are one- or two-sided<br>*Only common tests should be described solely by name; describe more complex techniques in the Methods section.*
[x]	[ ]	A description of all covariates tested
[x]	[ ]	A description of any assumptions or corrections, such as tests of normality and adjustment for multiple comparisons
[ ]	[x]	A full description of the statistical parameters including central tendency (e.g. means) or other basic estimates (e.g. regression coefficient) AND variation (e.g. standard deviation) or associated estimates of uncertainty (e.g. confidence intervals)
[x]	[ ]	For null hypothesis testing, the test statistic (e.g. F, t, r) with confidence intervals, effect sizes, degrees of freedom and P value noted<br>*Give P values as exact values whenever suitable.*
[x]	[ ]	For Bayesian analysis, information on the choice of priors and Markov chain Monte Carlo settings
[x]	[ ]	For hierarchical and complex designs, identification of the appropriate level for tests and full reporting of outcomes
[x]	[ ]	Estimates of effect sizes (e.g. Cohen's *d*, Pearson's *r*), indicating how they were calculated

Our web collection on statistics for biologists contains articles on many of the points above.

Software and code

Policy information about availability of computer code

Data collection	Localization data was collected on a home-built single molecule light field microscope platform based on an inverted Nikon TiU employing a 60x water-immersion objective. FCS measurements were made on a Zeiss LSM780 inverted confocal microscope using a 40× water objective. For full details, see Methods.
Data analysis	Image analysis was conducted in ImageJ (FIJI) with initial 2D localization of SMLFM data carried out in GSDC-SMLM v2.0 (PeakFit). 3D reconstruction, and the temporal grouping of 3D localizations into trajectories, was was conducted in Matlab R2021a using code that is available through Zenodo (DOI: https://doi.org/10.5281/zenodo.8190164). A maintained version of the 3D reconstruction code is available via GitHub (https://github.com/TheLeeLab/hexSMLFM). A custom Matlab code was used for fiducial correction of 3D SMLFM data. A custon Python code was used to conduct temporal grouping of localization data. Visualization of 3D point cloud data was carried out in ViSP (v1.0). The custom code used for ground-truth matching (PPV and sensitivity plots) was conducted in Matlab 2021a and is available via Zenodo with example simulated data (DOI: https://doi.org/10.5281/zenodo.8190164). FCS data was analyzed with PyCorrFit (v 1.17). DHPSF data was processed using DHPSFU (https://github.com/TheLaueLab/DHPSFU). Tetrapod PSF data was processed using ZOLA-3D (https://github.com/imodpasteur/ZOLA-3D).

For manuscripts utilizing custom algorithms or software that are central to the research but not yet described in published literature, software must be made available to editors and reviewers. We strongly encourage code deposition in a community repository (e.g. GitHub). See the Nature Portfolio guidelines for submitting code & software for further information.

Data

Policy information about availability of data

All manuscripts must include a data availability statement. This statement should provide the following information, where applicable:

- Accession codes, unique identifiers, or web links for publicly available datasets
- A description of any restrictions on data availability
- For clinical datasets or third party data, please ensure that the statement adheres to our policy

All B-cell receptor (live and fixed) and tubulin data generated in this study (accompanying Figs. 3--5) have been deposited on Zenodo [https://doi.org/10.5281/zenodo.8190164] (Ref: 47). Source data for Figs. 2--5 are provided with this paper.

Research involving human participants, their data, or biological material

Policy information about studies with human participants or human data. See also policy information about sex, gender (identity/presentation), and sexual orientation and race, ethnicity and racism.

Reporting on sex and gender	N/A
Reporting on race, ethnicity, or other socially relevant groupings	N/A
Population characteristics	N/A
Recruitment	N/A
Ethics oversight	N/A

Note that full information on the approval of the study protocol must also be provided in the manuscript.

Field-specific reporting

Please select the one below that is the best fit for your research. If you are not sure, read the appropriate sections before making your selection.

☒ Life sciences ☐ Behavioural & social sciences ☐ Ecological, evolutionary & environmental sciences

For a reference copy of the document with all sections, see nature.com/documents/nr-reporting-summary-flat.pdf

Life sciences study design

All studies must disclose on these points even when the disclosure is negative.

Sample size	No statistical analyses were performed in this paper and no statistical methods were used to predetermine sample size. Where relevant, sample sizes are reported in the figure legends and all experiments were performed independently at least three times to ensure results were repeatable.
Data exclusions	No data was excluded from analysis except in extreme cases where experiments failed (i.e. misalignment of microscope, issues with reagents).
Replication	Simulated single-molecule data consisted of 100 frames at each density and were repeated 3 times. Fixed BCR imaging experiments were repeated over 3 cells. Live BCR tracking experiments were repeated over 5 cells. Live BCR FCS experiments were repeated over 7 cells. Tubulin imaging experiments were repeated at least 3 times with consistent results. All attempts at replication were successful.
Randomization	Fields of view for imaging were chosen randomly. No biological comparisons were drawn.
Blinding	No blinding was performed in this study because it did not compare different biological systems.

Reporting for specific materials, systems and methods

We require information from authors about some types of materials, experimental systems and methods used in many studies. Here, indicate whether each material, system or method listed is relevant to your study. If you are not sure if a list item applies to your research, read the appropriate section before selecting a response.

Materials & experimental systems

n/a	Involved in the study	
☐	☒	Antibodies
☐	☒	Eukaryotic cell lines
☒	☐	Palaeontology and archaeology
☐	☒	Animals and other organisms
☒	☐	Clinical data
☒	☐	Dual use research of concern
☒	☐	Plants

Methods

n/a	Involved in the study	
☒	☐	ChIP-seq
☒	☐	Flow cytometry
☒	☐	MRI-based neuroimaging

Antibodies

Antibodies used	anti-α-tubulin (Abcam, ab7291) at 2.5 μg/mL
Validation	ab7291: Mouse monoclonal [DM1A] to alpha Tubulin.<br>> Species Reactivity: Mouse, Rat, Human<br>> Application: immunofluorescence, western blot, immunohistochemistry, and flow cytometry.<br>> Validation: https://www.abcam.com/products/primary-antibodies/alpha-tubulin-antibody-dm1a-loading-control-ab7291.html

Eukaryotic cell lines

Policy information about cell lines and Sex and Gender in Research

Cell line source(s)	HeLa TDS cells were gifted from MPI-CBG Technology Development Studio (TDS, Dresden, Germany). Primary mouse B cells imaged in this study originated from a single 8-12 week old, male, C57BL/6J mouse.
Authentication	Cell lines were not further authenticated.
Mycoplasma contamination	Cells tested negative for mycoplasma contamination by PCR.
Commonly misidentified lines (See ICLAC register)	No commonly misidentified cell lines were used.

Animals and other research organisms

Policy information about studies involving animals; ARRIVE guidelines recommended for reporting animal research, and Sex and Gender in Research

Laboratory animals	The primary B cells imaged in this study originated from a single 8-12 week old, male, C57BL/6J mouse.
Wild animals	N/A
Reporting on sex	No conclusions were drawn based on sex.
Field-collected samples	N/A
Ethics oversight	The organism in this study was not used as part of a regulated procedure (Schedule 1) and there is no protocol number because of this. No experiments were conducted on living animals.

Note that full information on the approval of the study protocol must also be provided in the manuscript.
